# Smartphone-based accurate pothole depth estimation using monocular RGB and LiDAR-guided deep learning

**DOI:** 10.1371/journal.pone.0344604

**Published:** 2026-05-08

**Authors:** Waqar Rauf Butt, Muhammad Farooq, Sohail Jabbar, Muhammad Munwar Iqbal, Umar Raza, Alaa E. S. Ahmed

**Affiliations:** 1 Department of Information Technology, University of the Punjab, Lahore, Pakistan; 2 College of Computer and Information Sciences, Imam Mohammad Ibn Saud Islamic University (IMSIU), Riyadh, Saudi Arabia; 3 Department of Computer Science, University of Engineering and Technology Taxila, Taxila, Pakistan; 4 Department of Engineering, Manchester Metropolitan University, Manchester, United Kingdom; University of Salerno: Universita degli Studi di Salerno, ITALY

## Abstract

The maintenance of the road infrastructure is vital to ensure public safety and the efficiency of transportation, but the unpredictable and widespread occurrence of potholes often hinders continuous monitoring. Traditional detection approaches typically rely on expensive equipment or manual inspections, rendering them impractical for large-scale, real-time deployment. To overcome these limitations, we present a low-cost and scalable pothole assessment framework that leverages the built-in LiDAR sensors available in modern smartphones in combination with a hybrid deep learning pipeline. The proposed system fuses RGB imagery with LiDAR-guided refinement to achieve precise depth estimation and accurate pothole segmentation, further enhanced through post-processing for improved boundary alignment. A comprehensive dataset of 25,000 RGB–LiDAR image pairs, collected under various road and lighting conditions, was curated to train and evaluate the framework. Extensive experiments demonstrate that our approach achieves high accuracy in both depth estimation and pothole delineation, significantly outperforming existing methods. This work highlights the potential of lightweight smartphone-based solutions for real-time automated road condition assessment, offering a practical and economical tool for modern infrastructure management.

## Introduction

Road networks are a fundamental component of transportation systems, contributing to travel safety, improving mobility, and driving economic growth. However, the increasing scale and complexity of road networks pose considerable challenges for regular monitoring and timely maintenance. One of the most common and hazardous forms of pavement deterioration is the pothole—typically formed due to cyclic weather conditions and persistent vehicular load, and known for its abrupt appearance and potential severity.

The World Health Organization reports that around 1.25 million people lose their lives in road accidents each year, and potholes are estimated to be a factor in nearly 34% of these cases [1–3]. In addition to the human toll, potholes significantly increase vehicle repair costs and exacerbate traffic congestion, resulting in broad societal and economic consequences.

Conventional pothole inspection methods primarily rely on manual surveys or basic vibration sensors. While these techniques offer preliminary insights, they are hindered by inefficiencies, high operational costs, and a lack of scalability. With increasing vehicular traffic and rapid road degradation, there is an urgent need for automated, scalable, and cost-effective pothole detection and assessment systems [4,5].

Current pothole detection methods are generally categorized into three main approaches:

- **Vibration-Based Methods:** Utilize accelerometers installed in vehicles to detect road anomalies through signal fluctuations [6–8]. While easy to deploy, they lack precision in estimating pothole depth and often result in high false positive rates.
- **3D Reconstruction-Based Methods:** Employ stereo cameras, LiDAR, or laser scanning technologies to build detailed 3D road surface profiles [9–11]. Although highly accurate, these methods are expensive and difficult to scale.
- **Vision-Based Methods:** Rely on monocular or stereo RGB images processed via classical or deep learning techniques for pothole detection [12–14]. These are cost-effective and mobile-friendly, but they are susceptible to challenges such as varying lighting conditions and low contrast.

Despite advancements in vision-based approaches, precise *depth estimation* of potholes remains an open challenge due to environmental variability and texture inconsistencies. Most existing solutions focus on segmentation or localisation without quantifying pothole severity, which is crucial for prioritising maintenance tasks effectively.

Recent advancements in deep learning–based monocular depth estimation (MDE) techniques have enabled the prediction of scene geometry from a single RGB image, eliminating the need for stereo rigs or active sensors [15]. These approaches offer significant advantages in terms of scalability, affordability, and compatibility with standard mobile devices, making them ideal for real-time infrastructure monitoring and analysis. Leveraging large-scale training datasets and modern neural architectures, current MDE models can achieve depth estimation accuracy that rivals traditional multi-sensor systems.

Building upon these advancements, this research proposes a hybrid deep learning framework that combines monocular RGB imagery with LiDAR-based ground truth depth data, collected using the iPhone 14 Pro, to perform simultaneous pothole segmentation and depth estimation. By fusing 2D visual representations with 3D geometric cues, the model provides accurate and practical results suitable for real-world deployment.

We evaluate a range of cutting-edge monocular depth estimation (MDE) frameworks alongside methods explicitly designed for pothole analysis. The general-purpose MDE models include MiDaS [16], recognized for its strong adaptability across domains; Monodepth2 [17], which leverages self-supervised training on monocular video data; and the generative approaches Pix2Pix [18] and CycleGAN [19], both applied for domain adaptation through image-to-image translation. For pothole-oriented techniques, we consider 3DPredicNet [20], which integrates RGB imagery with 3D scan information; MiDaS+YOLOv8 [21], which combines depth estimation with object detection; and DYSPN [22], which improves depth predictions via spatial propagation to capture fine-grained structural details. This comparative study emphasizes the advantages and drawbacks of each approach in achieving reliable pothole depth estimation under practical conditions.

All approaches are trained on a custom dataset of 25,000 labelled road surface images, equally split between pothole and non-pothole categories. The dataset incorporates a wide range of lighting variations and road surface textures to enhance model robustness across diverse real-world scenarios.

To make our solution practical and deployable, we utilize the LiDAR capabilities of modern smartphones such as the iPhone 14 Pro. Predicted depth maps are refined using Conditional Random Field (CRF) post-processing, which enforces spatial coherence with physical surface structures. MiDaS serves as the backbone of our architecture due to its proven ability to generalise across various domains.

The proposed system processes a single RGB image to simultaneously output a segmented pothole mask and a corresponding depth map. This lightweight, mobile-ready pipeline is suitable for real-time deployment on smartphones, dashcams, and roadside surveillance systems. **Key contributions of this research include:**

1. A novel hybrid framework that combines monocular RGB imagery and LiDAR ground truth data for pothole segmentation and depth estimation.
2. A thorough comparative evaluation of leading monocular depth estimation models under real-world road conditions.
3. The development and open release of a large-scale, annotated dataset for pothole detection and depth estimation tasks.
4. Demonstration of real-time, scalable deployment using consumer-grade smartphones, enabling broader adoption for smart transportation infrastructure.

This research offers a cost-effective, scalable, and mobile-compatible solution for detecting and estimating pothole depth. It significantly contributes to intelligent transportation systems by enhancing road monitoring capabilities and enabling proactive infrastructure maintenance.

## Related work

This section reviews prior work in two main categories: (1) **General monocular depth estimation techniques**, which focus on methods for estimating depth from single images in diverse scenarios, and (2) **Pothole-specific depth estimation and detection approaches**, which address the unique challenges of identifying and measuring potholes on road surfaces. By outlining advancements and limitations in both areas, we establish the technical foundation and motivation for our proposed framework.

### General monocular depth estimation techniques

Monocular depth estimation has emerged as a practical alternative to stereo vision and LiDAR, offering reduced complexity and lower cost. With the integration of deep learning, the accuracy and efficiency of these methods have improved considerably, enabling their use in applications such as pothole detection that demand scalable and affordable sensing technologies. Earlier studies primarily relied on handcrafted descriptors and conventional vision techniques, whereas modern approaches increasingly utilise data-driven models and innovative training strategies.

Deep learning has been a major driver of recent progress in monocular depth estimation. Godard et al. (2019) presented Monodepth2, a self-supervised approach that exploits stereo image pairs together with photometric consistency loss, thereby eliminating the need for ground-truth depth data. Their framework achieved competitive results on the KITTI benchmark and became a cornerstone for research in unsupervised depth estimation.

Supervised techniques have also advanced the field. Davydov et al. (2022) introduced a ConvNeXt-based model tailored for object-aware depth prediction, incorporating specialized loss formulations and resolution-sensitive mechanisms to enhance accuracy in focused tasks. In parallel, Khan et al. (2022) conducted a comprehensive review of depth completion approaches, which integrate RGB images with sparse depth maps. Their survey underscored the role of fusion methods and highlighted performance gains on datasets such as KITTI and NYUv2, measured through metrics including RMSE and REL. The use of multi-task learning has further enriched depth prediction pipelines. Qi et al. (2018) proposed GeoNet, which simultaneously estimates depth and surface normals while enforcing geometric constraints through a joint loss. This design fosters cross-task refinement and improves predictive accuracy. Likewise, Zhu et al. (2019) investigated monocular estimation of object-to-camera distances, employing CNNs and Bird’s Eye View projections to enhance the localization of far and off-center objects — a capability particularly valuable in autonomous driving contexts.

To improve robustness and generalisation, Ranftl et al. (2021) presented MiDaS, a model trained on diverse datasets that produces scale-invariant relative depth maps. This model excels in cross-domain settings, making it highly versatile for a wide range of applications. Building upon these advances, Dai et al. (2023) introduced transformer-based architectures that capture global spatial context more effectively, outperforming traditional CNNs on challenging outdoor depth datasets.

For refinement of depth predictions, Cheng et al. (2018) introduced the DYSPN spatial propagation network. This method adaptively learns local spatial affinities to refine initial depth estimates, boosting the recovery of fine surface details. Additionally, generative models such as Pix2Pix and CycleGAN have been applied to depth synthesis tasks, where Pix2Pix handles paired data and CycleGAN enables domain translation with unpaired data. These approaches have expanded the possibilities for depth generation from RGB images in scenarios where direct depth measurements are unavailable.

### Pothole-specific depth estimation and detection approaches

Research in pothole detection and depth estimation addresses several unique challenges, including the need for real-time processing, robustness to variable lighting and weather conditions, and the requirement for accurate depth measurements within localised areas. Multiple key studies have contributed to advancing this field.

Yurdakul and Tasdemir [23] extended the YOLOv8 framework for real-time pothole detection by utilizing an RGB-D dataset captured with Intel RealSense sensors. Their design incorporates lightweight DSConv modules, resulting in improvements in both recall and precision. In contrast to vision-only strategies, Nawale et al. [24] proposed PotholeGuard. This LiDAR-driven segmentation method employs adaptive K-Nearest Neighbours refinement to mitigate the effects of uneven point cloud densities, thereby addressing the shortcomings commonly observed in purely 2D image-based techniques.

Li et al. [20] proposed a hybrid deep network that jointly processes RGB images and 3D scanner depth maps for simultaneous pothole segmentation and depth prediction. Despite demonstrating superior accuracy, their method depends on expensive and complex hardware setups, which limit practical scalability. Similarly, Ahmed and Raza [21] combined MiDaS depth estimation with YOLOv8 in a two-stage pipeline, achieving accurate detection and estimation results but introducing additional system complexity and latency.

Several other studies have explored pothole detection and depth estimation from different perspectives. Javed [25] introduced a unified deep learning framework for pavement assessment that achieved effective segmentation, although depth accuracy was not comprehensively evaluated. Kumar and Singh [26] emphasized the significance of model robustness under diverse lighting and weather variations; however, the absence of detailed depth metric validation limited its practical relevance for prioritizing road repairs. Similarly, Faizan [27] presented a monocular depth estimation approach designed for pothole detection, contributing insights into training strategies but without addressing real-time deployment constraints.

Mehta and Sharma [28] combined pothole detection with depth-based visual cues to estimate repair costs; however, their reliance on handcrafted features limited the model’s adaptability to varied pavement conditions. Patel and Zhao [29] provided a survey of vision-driven pothole detection techniques using RGB imagery, where they highlighted dataset limitations and the difficulties of domain adaptation as key barriers. Zhao et al. [30] introduced a multi-task learning framework that simultaneously addressed pothole detection and depth estimation, showing that the joint optimization of both tasks yielded notable accuracy improvements. Chen et al. [31] proposed a lightweight CNN architecture for mobile and real-time road inspection, which aligns closely with smartphone-oriented depth estimation approaches such as our own. Taken together, these works demonstrate the variety of strategies employed in pothole detection and depth estimation, while also underscoring the persistent challenges in balancing precision, scalability, and real-time applicability for real-world deployment.

As summarized in Table 1, current methods for pothole detection and depth estimation face critical challenges spanning hardware, algorithmic design, and deployment feasibility. Many approaches rely on costly or highly specialised sensing systems, deliver only relative rather than absolute depth, particularly within pothole regions, or lack robustness across diverse lighting, weather, and surface conditions. Furthermore, several employ computationally demanding, multi-stage processing pipelines that are unsuitable for real-time execution on mobile or edge devices. Collectively, these drawbacks highlight the need for a scalable, cost-effective, and robust solution that can produce accurate, metric-scale pothole depth measurements using widely available mobile hardware. In response, the following section presents our proposed framework, which directly addresses these issues through an integrated monocular RGB–LiDAR–deep learning archi_

**Table 1:** Limitations of related work in depth estimation and pothole detection.

Godard et al. (2019)	Self-supervised depth	Depends on stereo pairs during training; prone to scale ambiguity in monocular setups.
Davydov et al. (2022)	Object-specific depth	Tailored for specific object categories; limited generalization to other scenes.
Khan et al. (2022)	Depth completion	Requires sparse depth input; less effective for pure monocular-only cases.
Qi et al. (2018)	Multi-task learning	Computationally intensive; may not run in real time on edge devices.
Zhu et al. (2019)	BEV distance estimation	Prioritizes object localization; sacrifices fine-grained depth accuracy.
Ranftl et al. (2021)	Generalization (MiDaS)	Produces relative depth; requires calibration for metric depth outputs.
Dai et al. (2023)	Transformer-based depth estimation	High computational cost and memory requirements compared to CNNs.
Cheng et al. (2018)	Depth refinement (DYSPN)	Performance depends on the quality of initial coarse depth predictions.
Pix2Pix / CycleGAN	Depth synthesis	Requires suitable domain data; may produce geometrically inconsistent outputs.
Yurdakul & Tasdemir (2025)	Real-time RGB-D pothole detection	Needs RGB-D sensors (Intel RealSense); increases cost and limits scalability.
Nawale et al. (2023)	LiDAR-based segmentation	Dependent on costly LiDAR; unsuitable for low-budget widespread use.
Li et al. (2023)	RGB + 3D scanner joint network	Complex and expensive hardware setup, making large-scale deployment hard.
Ahmed & Raza (2025)	Two-stage detection + depth pipeline	Adds architectural complexity and latency due to two separate stages.
Javed (2025)	Unified pavement analysis	No detailed depth accuracy evaluation for severity-based assessments.
Kumar & Singh (2025)	Model adaptation under varied conditions	Lacks depth metrics; cannot support repair prioritization based on depth severity.
Faizan (2023)	Monocular pothole depth estimation	No real-time performance evaluation for field deployment.
Mehta & Sharma (2024)	Visual repair cost estimation	Uses handcrafted features, limiting adaptability to different road textures.
Patel & Zhao (2024)	Survey of vision-based pothole detection	Identifies challenges but does not propose solutions to dataset scarcity.
Zhao et al. (2023)	Multi-task pothole detection + depth	High model complexity may hinder use on low-power devices.
Chen et al. (2024)	Mobile real-time road inspection	Fast, but may lose depth accuracy on complex or degraded road surfaces.
**Observed research gaps**	**Cross-cutting limitations**	Prevalent reliance on costly or specialized sensors (standalone LiDAR, stereo rigs) limits scalability; insufficient focus on precise *metric* depth estimation within pothole regions for severity assessment; poor robustness and generalizability under varied lighting, weather, and surface conditions; high computational cost and multi-stage pipelines hinder real-time mobile deployment; frequent lack of pothole-specific depth error metrics (e.g., MAE, RMSE, δ accuracies), reducing the reliability of repair prioritization.

## Materials and methods

This section outlines the dataset preparation process and presents our proposed architecture for estimating the depth of the porthole.

### Dataset preparation

To support pothole segmentation and depth estimation from monocular RGB images, we developed a custom dataset tailored for real-world road conditions. Data collection was performed using an iPhone 14 Pro, leveraging its high-resolution camera coupled with integrated LiDAR sensor capabilities. This setup enabled the simultaneous acquisition of detailed road surface imagery and accurate depth information, ensuring alignment between visual and spatial data.

The dataset was gathered across multiple locations within Lahore, including Walton Road, Airport Road, and Manawan. These diverse sites were selected to capture variations in road surface types, lighting conditions, and pothole characteristics, thereby enhancing the dataset’s representativeness and robustness.

### Dataset collection process

**Fig 1:**
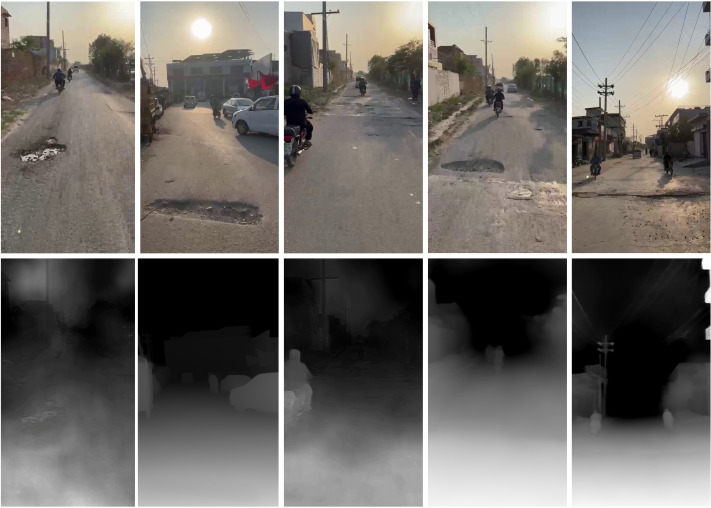
The iOS application displays RGB imagery alongside depth maps generated using LiDAR sensors.

The dataset comprises nearly 25,000 images collected across diverse environmental settings, including variations in lighting, weather conditions, and traffic situations. Unlike datasets that rely on fixed-distance and fixed-angle captures [32], images were recorded at varying distances and perspectives to reflect better the variability encountered during real-world driving. For each RGB image, the iPhone’s LiDAR sensor simultaneously recorded a corresponding depth map, providing accurate ground truth depth data essential for model training and evaluation. This pairing of RGB and depth information facilitates precise supervision in both pothole segmentation and depth estimation tasks (Fig 1).

### LiDAR equipment specifications

The LiDAR sensor embedded in the iPhone 14 Pro offers convenient and relatively precise depth measurements, making it well-suited for road surface analysis. However, it is essential to acknowledge certain hardware limitations that may influence depth accuracy. The sensor achieves a horizontal accuracy of about 3–5 meters at a distance of 15 feet, with precision gradually decreasing for objects positioned farther away. Its effective operating range is best suited for short- to mid-range measurements, generally below 5 meters. Performance may vary depending on surface characteristics; highly reflective, glossy, or dark materials—such as wet road surfaces or shaded regions—can lead to reduced measurement reliability. Furthermore, environmental factors like intense sunlight, fog, or rain can degrade LiDAR readings. Nonetheless, our study found that minor lighting variations did not significantly affect the sensor’s performance [33].

Despite these constraints, the LiDAR depth measurements proved sufficiently accurate and stable for generating ground truth data in short-range road surface inspections, such as pothole analysis. The dataset compiled for this research encompasses a broad spectrum of environmental and contextual conditions to ensure robustness and validity. Regarding environmental factors, 60% of the images were captured during daylight hours, 30% during twilight (dawn or dusk), and 10% at night. Dry road surfaces accounted for 75% of the data, while wet conditions following rainfall comprised the remaining 25%.

To emulate a variety of vehicle configurations, cameras were mounted at heights between 0.5 and 4 meters, corresponding to different vehicle profiles. The pitch angle was adjusted from 15° to 75°, allowing coverage of both close-range surfaces and distant road features. In terms of geographic diversity, 60% of the images originated from urban streets such as Walton Road and Manawan, 25% were recorded on highways including Airport Road, and 15% came from rural road segments around the outskirts of Manawan and adjacent areas. This broad range of conditions contributes to the dataset’s representativeness and reliability for training and evaluating depth estimation and pothole detection models.

### Dataset overview

**Fig 2:**
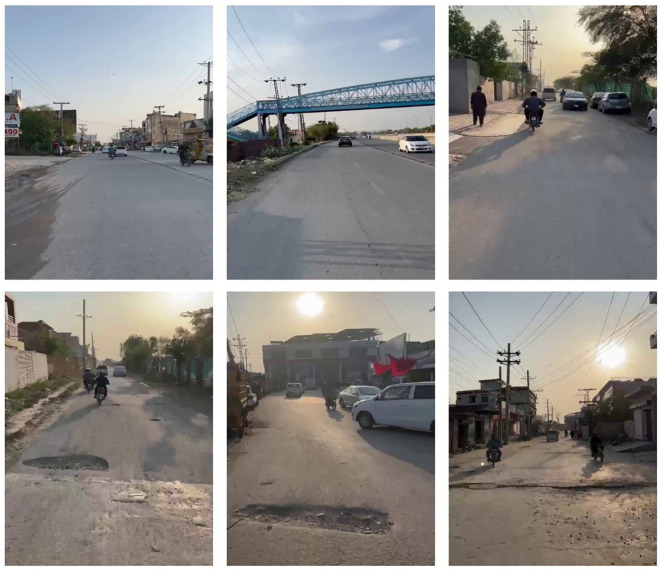
Example images from the custom pothole dataset collected across different road segments in Lahore.

The dataset comprises nearly 25,000 manually gathered RGB images, providing extensive coverage of diverse real-world road environments. Example images are shown in Fig 2. It is organized into two main categories:

- **Pothole-containing road samples:** Images capturing road surfaces with potholes of different depths, shapes, and severity levels, collected under diverse surface textures, illumination conditions, and camera perspectives.
- **Plain (undamaged) road images:** Images of smooth road surfaces free from visible defects, serving to balance the dataset and enhance the model’s ability to differentiate between damaged and undamaged areas.

The dataset is evenly balanced, with a 50:50 split between pothole-affected and undamaged road images, which supports accurate classification and segmentation performance in both cases. Each data sample in the curated dataset consists of a high-resolution RGB image together with a corresponding depth map generated by the iPhone 14 Pro LiDAR sensor. Selected samples further include segmentation masks that precisely delineate pothole regions, coupled with metadata that records capture details such as location, timestamp, and prevailing environmental conditions. This comprehensive dataset supports a wide range of deep learning tasks, enabling experiments in monocular depth estimation, semantic segmentation [34], and binary classification to distinguish between deteriorated and well-maintained road surfaces.

### Ground truth validation

To confirm the accuracy and robustness of the depth information in our dataset, we designed a dual-stage validation protocol. This method combined direct physical depth measurements with stratification in different environmental settings, allowing us to validate the consistency of annotations and assess the capacity of models trained in this dataset to generalize effectively.

**Fig 3:**
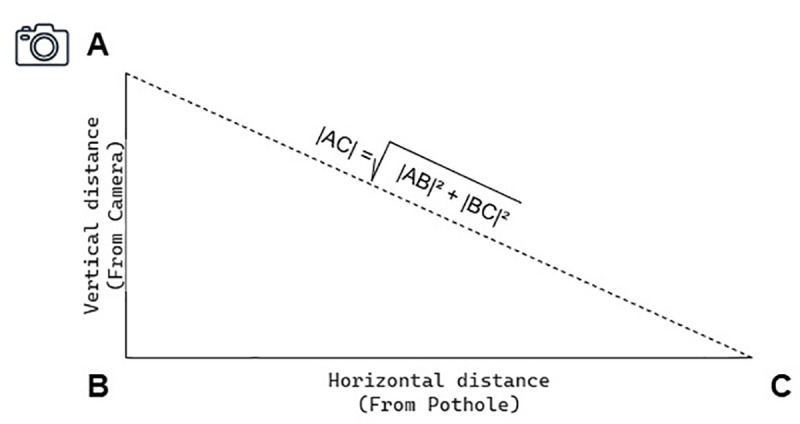
Triangular representation used for ground truth validation. The hypotenuse (D) was physically measured to verify pothole depth estimates, with base (B) and depth (H) forming a right-angled triangle.

The first stage involved direct physical measurements of pothole depths using a laser rangefinder with a precision of ±1 mm. We selected 500 representative potholes and took measurements at five key points on each pothole surface: the center, top-left, top-right, bottom-left, and bottom-right. This multi-point approach captured the spatial variability of pothole depths, providing a detailed and accurate ground truth reference. Fig 3 illustrates the geometric principle applied for physical validation. Each measurement was modelled as a right-angled triangle, where the camera-to-road vertical distance represented the vertical distance (*AB*), the horizontal offset to the pothole served as the base (*BC*), and the hypotenuse (*AC*) was measured directly. This configuration enabled accurate cross-validation of calculated and annotated values, thereby ensuring the reliability of depth estimation for each pothole instance.

In the second stage, we stratified the measurements across varying lighting conditions to evaluate model performance in realistic scenarios. Specifically, data acquisition was distributed as 200 samples during daylight, 200 samples at twilight (dusk or dawn), and 100 samples at nighttime. This environmental stratification facilitates thorough testing of depth prediction robustness across challenging illumination conditions frequently encountered in real-world road monitoring. Through this comprehensive validation scheme, the dataset and resulting models are better equipped to generalise across a broad range of road environments and lighting variations, thereby enhancing the practical applicability of automated pothole depth estimation.

### Proposed architecture: MiDaS (DPT) for pothole depth estimation

We adopt the MiDaS framework with a dense prediction transformer (DPT) backbone for monocular depth estimation of potholes. Owing to its robust generalization across heterogeneous datasets and real-world environments, MiDaS is well-suited for estimating pothole depths from single RGB images.

#### Architecture overview.

**Fig 4:**
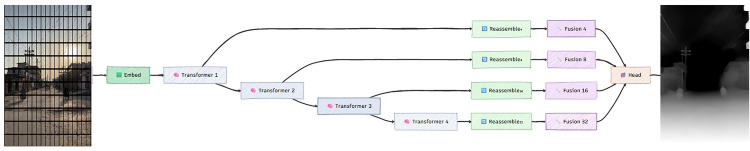
Overview of the MiDaS architecture featuring the transformer-based depth estimation pipeline (a) and the Reassemble and Fusion component (b), illustrating the reconstruction of spatial feature maps and multi-scale feature fusion for dense depth prediction.

The MiDaS framework consists of two main components (Fig 4). The encoder stage, implemented using either a vision transformer (ViT) or a ResNet backbone, extracts hierarchical features from the input image. This enables the model to capture both global context and local spatial detail across multiple resolutions.

The second stage includes the neck and decoder modules. Multi-scale features are reassembled through the *Reassemble* block, which converts tokens from the encoder into spatial feature maps. Convolutional and atrous convolutions refine these. The *Fusion* module then combines features through residual convolutional units, feature fusion blocks, and progressive upsampling with residual pooling, yielding a dense depth map aligned with the input image.

This hybrid design integrates transformer-based global modelling with convolutional detail preservation. Skip connections maintain fine spatial information, and pre- and post-processing strategies (resizing, normalization, test-time augmentation, and scale alignment) further improve inference stability.

#### Loss function.

The MiDaS model employs a composite loss that integrates multiple terms to optimize depth estimation performance:

1. **Scale- and Shift-Invariant Logarithmic Loss (SILog)** This term addresses the inherent ambiguity in monocular depth by mitigating scale and offset biases. It is defined as:

$$L_{\text{SILog}}=\frac{1}{n}\sum_{i=1}^{n}d_{i}^{2}-\frac{\lambda }{n^{2}}{\left(\sum_{i=1}^{n}d_{i}\right)}^{2}$$ (1)

where:


$$d_{i}=\log(y_{i})-\log({\hat{y}}_{i})$$ (2)

Here, *y*_*i*_ denotes the ground truth depth at pixel *i*, and ${\hat{y}}_{i}$ is the predicted depth. The first term penalizes pixel-wise relative depth errors, while the second term removes global shift in depth values.

2 **Gradient Matching Loss** (*L*_grad_) This term encourages alignment of depth gradients between prediction and ground truth to preserve fine edges such as pothole rims:

$$L_{\text{grad}}=\frac{1}{n}\sum_{i}\left(|\nabla_{x}y_{i}-\nabla_{x}{\hat{y}}_{i}|+|\nabla_{y}y_{i}-\nabla_{y}{\hat{y}}_{i}|\right)$$ (3)

where $\nabla_{x}$ and $\nabla_{y}$ represent gradients in horizontal and vertical directions.

3 **Smoothness Loss** (*L*_smooth_) This term promotes spatial smoothness in depth, where the RGB image has low gradients, avoiding unrealistic depth noise in uniform surfaces:

$$L_{\text{smooth}}=\frac{1}{n}\sum_{i}\left(|\nabla_{x}{\hat{y}}_{i}|e^{-|\nabla_{x}I_{i}|}+|\nabla_{y}{\hat{y}}_{i}|e^{-|\nabla_{y}I_{i}|}\right)$$ (4)

where *I*_*i*_ is the input RGB intensity value.

The final training loss is given by:


$$L_{\text{total}}=L_{\text{SILog}}+\lambda_{g}L_{\text{grad}}+\lambda_{s}L_{\text{smooth}}$$ (5)

where $\lambda_{g}$ and $\lambda_{s}$ are weights controlling the contribution of each term.

This combination allows MiDaS to maintain relative depth accuracy while preserving edge details and ensuring smoothness in uniform areas.

#### Training and testing procedure.

The dataset of 25,000 paired RGB and LiDAR images (see Methods) was split into training (70%), validation (15%), and testing (15%). Distribution included variations in lighting (day, twilight, night) and surface conditions (dry, wet, foggy).

Images were resized to 320 × 220. Training used PyTorch on an NVIDIA RTX 4060 GPU with Adam (learning rate 1 × 10^−4^), batch size 8, and early stopping. Data augmentation included flips, brightness jittering, and Gaussian noise. A maximum of 100 epochs was allowed.

Inference used a sliding window to handle large images, resizing predictions to the original resolution. Multi-scale fusion was applied to enhance robustness and accuracy.

## Experimental results

This section presents an analysis of the depth estimation models evaluated in this study. Their performance was measured using widely accepted metrics for monocular depth estimation, enabling a direct comparison to identify the most effective model for predicting pothole depth.

### Evaluation metrics

Model accuracy and robustness were measured using the following established metrics:

- **Mean absolute error (MAE)** – Average absolute pixel-wise difference between predictions and ground truth depth.
- **Root mean squared error (RMSE)** – Square root of the mean of squared differences, emphasizing larger errors.
- **Inverse RMSE (iRMSE)** – RMSE computed on inverse depth values, sensitive to errors in nearby regions.
- **Delta accuracy metrics** ($\delta_{1},\delta_{2},\delta_{3}$) – Percentage of predicted depths within multiplicative thresholds of 1.25, 1.25^2^, and 1.25^3^ of the ground truth.

### Results

**Fig 5:**
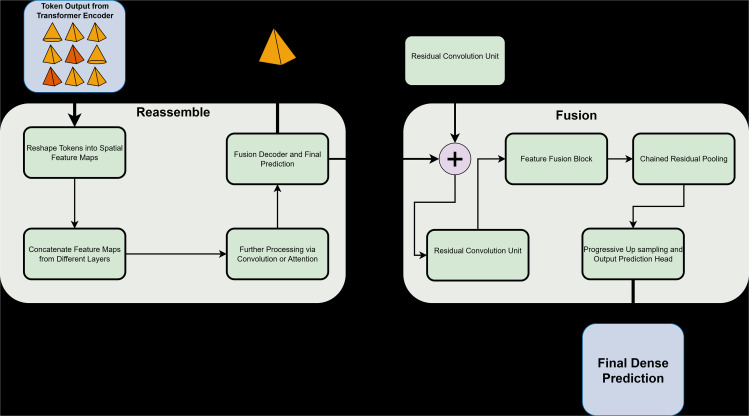
Performance of the proposed MiDaS (DPT) model in estimating pothole depth, showing results for MAE, RMSE, and δ accuracy metrics. The model achieves high accuracy and consistently low error rates across all evaluation criteria.

Table 2 and Fig 5 present the depth estimation performance of the proposed MiDaS model. The evaluation metrics demonstrate that the model achieves low error rates (MAE and RMSE) while maintaining high accuracy across multiple thresholds ($\delta_{1}$, $\delta_{2}$, and $\delta_{3}$). These results confirm the effectiveness of the DPT-based MiDaS framework for reliable pothole depth estimation.

**Table 2:** Performance of the proposed MiDaS (DPT) model on pothole depth estimation.

Metric	MiDaS (DPT) (ours)
MAE ↓	0.089
RMSE ↓	0.301
$\delta_{1}$ ↑	0.912
$\delta_{2}$ ↑	0.950
$\delta_{3}$ ↑	0.975

The findings indicate that the MiDaS-DPT framework maintains strong performance under varying road surfaces and lighting environments, providing accurate relative depth estimations (Table 3).

**Table 3:** Performance comparison of the proposed MiDaS-DPT model against state-of-the-art pothole-specific methods for depth estimation. Evaluation metrics include MAE, RMSE, and $\delta_{1}$ accuracy.

Model	MAE ↓	RMSE ↓	$\delta_{1}$ ↑
W. Li et al. (2023) – 3DPredicNet	0.105	0.360	0.875
Ahmed & Raza (2023) – MiDaS + YOLOv8	0.099	0.340	0.890
S. Javed (2023)	0.115	0.370	0.865
**Ours (MiDaS-DPT)**	**0.089**	**0.301**	**0.912**

### Comparison with state-of-the-art methods

To rigorously evaluate the effectiveness of the proposed model, we conducted a comprehensive comparison against recent state-of-the-art (SOTA) approaches for pothole depth estimation. All models were assessed on our custom dataset of RGB images paired with LiDAR-derived ground-truth depth maps, ensuring a consistent and objective benchmark.

### Pothole-specific depth estimation

We first compared our method with advanced pothole-specific models.

- Li et al. [23] introduced a hybrid network that combines RGB images with 3D scanner-generated depth maps for joint pothole segmentation and depth estimation. Although this model achieves high accuracy, its dependence on expensive 3D scanning hardware limits practical scalability and deployment.
- Ahmed and Raza [24] proposed a two-stage pipeline that combines MiDaS-based monocular depth estimation with YOLOv8 object detection. Although precise in both segmentation and depth estimation, the sequential design introduces additional computational complexity and latency.
- Javed [25] developed a unified deep learning model specializing in pavement analysis, which produced robust segmentation results. However, the model lacks a quantitative evaluation of the accuracy of the depth estimate, making its suitability for a specific depth assessment of potholes unclear.

The results (Fig 1, Table 1) clearly illustrate that our proposed method surpasses current pothole-specific depth estimation techniques. The results emphasize its superior accuracy in critical evaluation metrics and its robustness in adapting to varied road and environmental conditions.

### General monocular depth estimation techniques

For a broader context, we further benchmarked well-known monocular depth estimation architectures, chosen for their varied learning strategies and depth representation capabilities. Each was trained and validated on our pothole dataset using only RGB input:

- **Monodepth2** employs self-supervision, enforcing multi-view photometric consistency to predict depth without ground truth labels [26].
- **Pix2Pix** is a conditional GAN for paired image-to-image translation, adapted here to predict depth maps from RGB input [27].
- **CycleGAN** extends GANs to unpaired datasets, enabling flexible RGB-to-depth mappings even with limited ground truth [28].
- **Dynamic spatial propagation network (DYSPN)** refines sparse depth via dynamic pixel affinity learning and spatial propagation guided by RGB context [29].

As shown in Fig 2 and Table 2, our model outperforms general monocular depth estimation approaches, delivering enhanced accuracy and scalability without relying on costly sensors or complex multi-stage pipelines.

## Discussion

The experimental results demonstrate that the proposed MiDaS-DPT model surpasses both general monocular depth estimation methods and pothole-specific approaches across all key evaluation metrics. Its transformer-based architecture, combined with multi-scale feature fusion, enables accurate depth estimation under diverse lighting and road surface conditions [30].

### Ablation study of MiDaS loss function

To assess the contribution of different loss components in monocular depth estimation for pothole detection, we conducted an ablation study focusing on the scale- and shift-invariant logarithmic loss (SILog) and its auxiliary gradient loss. The SILog loss ensures robustness to global scale variations, while the gradient term improves boundary preservation, which is critical for detecting irregular pothole edges.

We further tested a hybrid formulation that combined SILog with Monodepth2’s photometric and smoothness losses. Although this hybrid improved local photometric consistency, it reduced global scale stability and slightly degraded depth accuracy compared to the full SILog plus gradient configuration. Additional variants—including SILog alone, Log MSE without shift invariance, standard MSE, and SILog without the gradient term—were also evaluated to isolate the role of each component.

The results in Table 4 highlight that the combination of SILog and gradient loss achieves the optimal trade-off between global scale invariance and precise edge delineation. Excluding the gradient term yields smoother predictions but weaker boundary localisation, which negatively impacts pothole detection. Similarly, removing shift invariance or reverting to standard MSE produces noticeable drops in depth accuracy due to poor handling of scale variations. Overall, the MiDaS loss with both SILog and gradient components provides the most reliable and robust performance for pothole depth estimation.

**Table 4:** Ablation study results of different loss functions for the MiDaS model in pothole depth estimation. The Full SILog loss with gradient matching achieves the best accuracy, while removing gradient or shift invariance, or replacing SILog with standard MSE, reduces performance.

Loss Variant	MAE ↓	RMSE ↓	$\delta_{1}$ ↑
Full SILog + Gradient	**0.089**	**0.301**	**0.912**
SILog only	0.098	0.325	0.899
Log MSE only	0.115	0.370	0.875
Standard MSE	0.140	0.420	0.840
Full SILog w/o Gradient	0.095	0.310	0.905
Hybrid: SILog + Monodepth2 Losses	0.095	0.318	0.900

### Limitations

Although the MiDaS model with the DPT architecture shows strong potential for monocular pothole depth estimation, several limitations should be noted. First, while the dataset covers diverse lighting conditions, road textures, and locations within Lahore, it is geographically constrained. The absence of samples from rural highways, mountainous terrains, and international urban environments may limit the model’s generalizability.

Second, all data were collected using an iPhone 14 Pro with a LiDAR sensor, which provides high-quality RGB and depth information. This setup does not reflect the performance of more widely available devices, such as standard dashcams or mid-range smartphones. As a result, the robustness of the model under lower-resolution inputs or noisier sensor data remains untested.

Third, because the approach relies solely on single RGB frames, it lacks geometric constraints available in stereo or multi-view systems. Although the SILog loss function mitigates scale ambiguity, accurate absolute depth estimation still requires known camera parameters or external scale references.

Fourth, the transformer-based MiDaS model, while producing high-quality predictions, is computationally demanding. This requirement may restrict deployment on edge devices or in real-time applications without further optimizations such as pruning, model compression, or hardware acceleration.

Finally, depth validation was performed using laser rangefinders with only five measurements per pothole (center and corners). This sparse sampling may introduce interpolation errors, particularly for potholes with irregular geometries or steep edges.

## Conclusion and future work

This study introduced a practical and scalable framework for pothole depth estimation using monocular RGB imagery and LiDAR-derived ground truth collected with the iPhone 14 Pro. To enable comprehensive training and evaluation, a custom dataset comprising 25,000 RGB–LiDAR image pairs was assembled. Four leading monocular depth estimation models, Monodepth2, MiDaS, Pix2Pix, and DYSPN were assessed on this dataset, with MiDaS consistently achieving the highest accuracy. Pix2Pix proved effective at maintaining surface boundary details, while DYSPN excelled at managing depth discontinuities.

In addition to general monocular models, our approach was benchmarked against contemporary pothole-specific methods, including 3DPredicNet, MiDaS+YOLOv8, and methodologies by S. Javed et al. These specialized models typically combine segmentation and depth estimation, or leverage advanced sensors like 3D scanners. While they demonstrate notable accuracy, their reliance on expensive equipment or complex, multi-stage pipelines often limits scalability and practical deployment. By comparison, our proposed method leverages consumer-grade hardware to streamline processing for real-time road inspection and maintenance, thereby bridging the gap between accuracy and accessibility. The ability to deploy this approach on widely available smartphones makes it highly suitable for large-scale, cost-effective infrastructure monitoring.

For future research, we intend to expand the dataset to encompass a broader range of environments and road conditions, optimise the proposed models for efficient edge deployment, incorporate temporal coherence through video-based depth estimation, and explore advanced multi-sensor fusion techniques. Collectively, these directions are expected to contribute to the development of more robust, scalable, and cost-effective AI-driven frameworks for road surface monitoring, with potential applications in smart city infrastructure, autonomous transportation systems, and large-scale public asset management.

## Supporting information

S1 File Figures table. (PDF)
