# Supplementary material for: Smartphone-based accurate pothole depth estimation using monocular RGB and LiDAR-guided deep learning
Source: PLoS One. 2026 May 8;21(5):e0344604. doi: 10.1371/journal.pone.0344604 (PMC13155672; doi:10.1371/journal.pone.0344604)
Supplement: S1 File — (PDF) [file pone.0344604.s001.pdf]

Figure Submission Table for PLOS ONE

Fig #	Caption (Overleaf)	File to Upload	Notes / Combined
1	The iOS application displays RGB imagery alongside depth maps generated using LiDAR sensors.	Fig1.tif	Single image
2	Example images from the custom pothole dataset collected across different road segments in Lahore.	Fig2.tif	Single image
3	Triangular representation used for ground truth validation. The hypotenuse (D) was physically measured to verify pothole depth estimates, with base (B) and depth (H) forming a right-angled triangle.	Fig3.tif	Single image
4	Overview of the MiDaS architecture; (a) transformer-based depth estimation pipeline, (b) Reassemble and Fusion component illustrating reconstruction of spatial feature maps and multi-scale feature fusion.	Fig4_a.tif, Fig4_b.tif	Two separate subfigures (a) & (b)
5	Performance of the proposed MiDaS (DPT) model in estimating pothole depth, showing results for MAE, RMSE, and δ accuracy metrics. The model achieves high accuracy and consistently low error rates across all evaluation criteria.	Fig5.tif	Single image
